# Epidemic Cholera—Its History and Ætiology

**Published:** 1832

**Authors:** 


### Art. VII *Epidemic Cholera—Its History and Ætiology.*

### An Eclectic Review.

We are incredulous as to the assertion, that the present invasion of Epidemic Cholera has destroyed fifty millions of human beings, but of the fact, that it has spread eastwardly from the Delta of the Ganges to the Islands of the Pacific ocean; and, at length, as if in quest of new victims, turned westwardly and traversing Europe, has crossed the Atlantic, there can be no dispute. About the 8th of June, 15 years after its appearance on the jungly banks of the sacred river, we find it engaged in the work of death on the shores of the St. Lawrence; and threatening to ascend into the region of inland seas, which give sublimity and beauty to the interior of the new world. That it will descend upon the valley of the Mississippi, and carry dismay and temporary desolation throughout the beautiful and happy regions of the West, let us not presume to doubt.

The question has been often asked, is not this disease identical with the cholera morbus of the countries which have been scourged? It *may* be ; but if so, some cause has been acting, progressively, to aggravate the ordinary epidemic, into a degree of malignity which it never displayed before. For ourselves, we think it bears to cholera morbus, a relation similar to that of the influenza to a common cold from changes of weather.

But will not the disease be modified in a favorable way, by the climate or state of society in the United States? It is impossible to answer this question in a satisfactory manner. The reports from Montreal and Quebec represent it as still more fatal than it ever was on the banks of the Ganges, the Volga or the Thames; but the shores of the St Lawrence present a floating and indigent population, among which it could not fail to revel; and when it reaches a country comparatively exempt from want, it *may* assume a milder character ; at present however, we can only indulge in conjectures. Let us proceed to its history.

The present Epidemic seems to have commenced, in the year 1817, in the Delta of the Ganges. The first accounts of it were from the City of Jessore 80 or 100 miles N. E. of Calcutta, in the month of August ; but it was afterwards ascertained, that it prevailed in some parts of Nuddecca, as early as the month of May. Before winter, it laid waste a great number of towns along the Ganges, and in various parts of the peninsula of Hindoostan, including Calcutta, where it manifested itself early in August. Thus during the same unpropitious summer, the Epidemic broke out, almost simultaneously, in different and distant places.

It is not our design to trace its progress from year to year over Asia, till it entered Europe in 1829; but we may remark, that for a period of 12 years, it prevailed constantly in some part of Asia. Extending eastwardly, it invaded Siam, Cochin China, China proper, and even reached Pekin in 1825. Leaving the

Continent, it visited Java, the Phillipines and many other Islands, extending to Banda, in the Pacific Ocean, in longitude 128 deg. E. To the south of the Indian peninsula, it assailed the island of Ceylon and even reached Bourbon, in the southern hemisphere. To the west, it passed the Indus, meandered the northern shore of the Persian Gulph, ascended the Tigris and Euphrates, and arrived at Antioch, Aleppo and other places, near the eastern extremity of the Mediterranean. Arabia seems to have resisted its progress to the south, but to the north, different parts of the Persian Empire, were signally scourged, and the pestilence spread quite to the sources of the Ganges and Indus. The Himmalah mountains, alone seemed to stay its progress.

Before proceeding to trace the course of the Epidemic in Europe, let us enumerate some of the facts connected with its Asiatic History.

1. As already stated it appeared in various places, nearly at the same time.

2. After visiting a town or city it frequently within a few months, or in some subsequent year, returned upon it, with equal or greater violence.

3. It often left towns and narrow slips of country unaffected, prevailing quite around them; and sometimes attacked them subsequently, when adjoining places were healthy.

4. It affected both town and country, but most of the reports concerning it, are drawn from the former.

5. In towns, it has generally raged with the greatest virulence, where the population was most dense. It has sometimes confined itself to one side of a street, and often left considerable portions of a city untouched, although free intercommunication existed.

6. In several instances it has seemed to be introduced into towns by ships or caravans of travellers; at least has commenced soon after their arrival, and spread rapidly, when it did not exist there before.

7. It has frequently arisen in towns, which had not had any intercourse, with places in which the disease prevailed. It has appeared in ships at sea, which for a long time before had not been exposed to it on land.

8. It has constantly been disposed to assail large assemblages of people.

The armies of Asia have been peculiarly obnoxious to it. When marching they have been suddenly and mortally assailed. Whenever the army has dispersed in small bands, the disease has ceased. One wing of an army has sometimes been affected while the other continued healthy. A detachment of troops has sometimes, while affected with the disease, rejoined the main army, without communicating it. A marching army has been suddenly attacked and as suddenly relieved, by changing its place of encampment. In marching, the disease has appeared and disappeared, in an army, two or three times, according as it prevailed or did not prevail, in the districts over which the march was directed.

10. All over Asia, it has prevailed most on the margins of rivers, near their mouths, and in other damp and vaporous localities.

11. It has prevailed on one side of a river for some time, and then crossed to the other.

12. Sometimes it has attacked the inhabitants of dry and elevated places—It has even ascended to the height of 5 or 6,000 feet.

13. It has often traversed countries in the direction of their great roads, but to this there are many exceptions.

14. It has equally affected the Hindoos, the Europeans resident in India, and the natives of that country, descended from European parents.

15. Every where in Asia it has fallen most heavily upon the alarmed ; the poor; the aged and those enfeebled by previous disease or great exertions; those who lodged in confined, damp, and dirty places; the intemperate; the half famished, and those who exposed themselves to the night air.

16. In India it has generally affected adults much more than children; and men more than women; in some places, however, the reverse of the latter has occurred.

17. Cholera patients have often been received into the general hospitals, without communicating the disease to the patients already there, on the other hand, the disease has sometimes seemed to spread in this manner.

18. Physicians and nurses, have been observed to be less subject to attacks, than other people; but they have occasionally been affected, and in a few instances have appeared to contract the disease from such exposure.

19. The disease has sometimes prevailed throughout all seasons of the year, but generally has been suspended in winter.

20. In most instances, its invasions has been sudden, and like other epidemics, it has been most fatal at the onset. The subsequent cases have become milder and more manageable. At length it has disappeared, leaving, in many places, a large number untouched. The proportion of those affected by it has varied exceedingly in different towns. Its duration has sometimes not exceeded a fortnight, but in other cases it has continued for two or three months. In general, the more dreadful the energy of the invasion, the shorter has been its duration.

21. In various parts of India and Persia, earthquakes have occurred several times since the Epidemic first arose. In one instance, it appeared with great mortality in a body of troops, immediately subsequent to a violent tornado. At other times, it has ceased after thunder storms.

22. The winds have not seemed either to hasten or retard the march of the Epidemic.

23. It has *commonly* made its attacks in the latter part of the night.

24. In many places, mortal diseases in the domestic animals, both birds and quadrupeds, have been observed to precede, accompany or follow, its visitations upon the inhabitants.

25. It has not ceased in Asia since its introduction into Europe.

Such are the principal and the most important results of the observations made in Asia. Let us now turn our attention to Europe.

For 12 years the Epidemic ravaged India, Persia, and Syria, without passing the borders of the Caspian sea—and the summit of the Ural mountains. In the month of August 1829 it entered Russia. The town of Orenburg situated on the right bank of the river Ural, in latitude 51 degs. N. and longitude 72 degs. E. has generally been regarded as the place first affected. We shall transcribe from the elaborate report of a committee of the French Academy of Medicine, made to the government in July 1831, what seems to be an impartial digest of the facts in this case.

"Behold then the Cholera primarily established in a city which scarcely presented any general cause of insalubrity. It is true, that the season, immediately preceding that in which the epidemic appeared, had been irregular, inconstant, and marked by great atmospheric vicissitudes; the humidity especially had been great and sudden, succeeding prolonged heats and extreme drought.

It is also true, that a considerable quantity of fruits of an indifferent or bad quality had been gathered in this year, and the inhabitants had freely eaten of unripe water melons and cucumbers.

It is also true that the *koumiss*, a species of drink made from fermented mare's milk, had failed, and still more the *knout*, an analogous but better drink made from the milk of the sheep and the cow.

But already, in antecedent years, had all these anti-hygienic conditions been frequently presented, in the same degree, and in similar combinations, yet without the least appearance of epidemic Cholera.

Let us further add with respect to fruits, that subsequently to their absolute interdiction, and notwithstanding the rigid measures adopted by the public authorities to effect their total exclusion from the city, the disease increased with greater activity and counted a greater number of victims.

The Cholera having appeared then at Orenburg notwithstanding the salubrity of this city, it became necessary to seek elsewhere for its origin; the epidemic reason not being as yet presented to meditative minds, or rather this reason alone was not deemed satisfactory.

It is in this state of the question that the origin of the disease has been attributed to an importation imputed, by some, to the caravans arrived from Boukaria and Khiva, and by others, to the commercial relations established with the neighbouring hordes of Kirguis-Cossacks.

The Kirguis, a wandering and half-savage people, encamped in the immense *steppes*, beyond the Ural mountains, being in constant commercial intercourse with Turkestan, Boukaria, and Khiva, have long been in the habit of furnishing Orenburg with sheep, felt, camlets, &c. Now, when the Cholera appeared at Orenburg, we know that the epidemic was extending its ravages in Chorazan, in Asia and Persia.

Let us, however, examine both assertions, that is to say, the importation of the Cholera by caravans and by the Kirguis.

On the 26th of August, 1829, at nine in the morning, surgeon-major Smirnorff detected the Cholera-Morbus in Andre Yvanoff, a soldier of the 3d battalion of the line then in garrison at Orenburg, who was carried to the military hospital at Orenburg, where he died at about the expiration of twelve hours from the commencement of the disease.

From the 26th of August to the 9th of September, no other person in Orenburg was attacked with the Cholera.

On the 9th of September, at 11 o'clock in the night, another soldier, of the same battalion of the line, likewise attacked with this malady, was brought to the military hospital. He died towards five o'clock in the evening of the following day.

The day after, September 10th, two other patients were sent to the hospital, one of whom died, the other was cured. In both these cases the disease had progressed with less rapidity than in the two preceding.

On the 11th, a soldier of the same battalion was attacked and carried to the hospital; then on the 14th, a soldier of the battalion of invalids; again, on the 16th, two other soldiers of the same battalion, and, finally, on the 17th, two non-commissioned officers.

During the whole of this time, and until the 18th of September, only two individuals among the citizens of Orenburg had been seized with the malady; both of whom died. One was an excise officer, the other a tradesman.

The commerce of Orenburg, a city altogether commercial, has for a long time been maintained in frequent, extensive, and important relations with Asia, India, and Persia: it is at least since 1813 that the city of Orenburg has been in the habit of admitting annually the caravans coming from Kiachta and Boukaria: now, the Cholera reigned epidemically in these countries during the month of August, 1817, and it was not until August, 1829, that it first made its appearance at Orenburg.

The caravans from Boukaria and Khiva arrived in July (the 20th at the latest) at Orenburg, were admitted into the Court for Strangers, after sanitary visits and inspection, but the Cholera was not manifested until the 26th of August.

Convoys making a part of these caravans, had entered about the same time into the fortresses of Orstia and Troitzka, yet the Cholera did not appear in these two fortresses.

The guards and camel-drivers, hired by these caravans in one place and in another throughout their route, have entirely escaped the disease.

It must also be noted that these Asiatic traders loaded and unloaded their camels during their journey; that whenever they arrived at an *aoula* or encampment of Kirguis, they made numerous exchanges of merchandise for provisions of every sort, which consequently rendered it necessary to unpack the bales in which they were contained: yet, notwithstanding these different operations, there has not been a single instance in which Cholera was communicated.

From the 26th of August, the day in which the first patient, the soldier Yvanoff, was attacked by the disease, and who died with it on the same day, until the 9th of September, there was not a single individual seized with the Cholera at Orenburg.

The Cholera suddenly makes its appearance among the poorer class of people, in individuals worn down by hard labour and debilitated by extreme want, It was not first observed among custom-house officers, in continual communication with the suspected Kirguis and in constant contact with the suspected articles of merchandize. It has not attacked the merchants who purchased camlets, felts, and furs ; it has respected easy or rich people who carried them. The traffic-market of Orenburg, situated at the distance of three *versts** from the city, on the right bank of the Ural, where, during the whole summer and one half of the autumn, the Russian and Asiatic merchants reside and exchange their merchandize with that of the Kirguis, it would seem ought to have been the first theatre on which the disease appeared ; yet, notwithstanding, it was in a lesser proportion and at a later period that these quarters were attacked.

The Cholera had existed a long time in the city of Orenburg, when it broke out in the badly constructed fauxbourgs which constitute the boundaries of it.

Now it was not until the 28th of September that information was received of the first appearance of the Cholera in the environs of Orenburg ; and, surprising circumstance! whilst the villages nearest to the city, having intimate relations and constant communication with it, were altogether exempt from the Cholera ; whilst the fortresses adjacent to Orenburg and situated in the line of the two principal roads which terminate at this place, preserved their habitual salubrity,—during this time, the Cholera exhibited its accustomed ravages in villages at some distance from Orenburg, and also in a fortress situated at more than a hundred versts from this city, which had maintained but very little communication with it.

The villages lying east from Orenburg, between the Ural mountains and the city, but beyond the latter, in the line of communication of Asia with Orenburg ; these villages, Nieginka and Kaminoe for instance, although built along the upper Ural and having free intercourse every hour, as well as every day, with the city, have not been invaded by the malady.

In the military hospital of Orenburg, the Cholera began on the 26th of August, and did not cease its reign until the 20th of November ; the number of persons attacked with the disease was two hundred and ninety-nine, of which number two hundred and twenty were cured and seventy-nine died: we have stated above that the garrison consisted of six thousand men.

In the city of Orenburg, the disease did not commence until the 15th of September ; and on the 20th of November there was not, either in the city, or its suburbs, a single patient with the Cholera. Of the entire population of the city and its fauxbourgs, which is estimated at seven thousand, there were eight hundred and one patients, out of which number six hundred and eighty were cured and one hundred and one died.

*About two miles ; a *verst* being nearly two thirds of an English mile.— Tr.

Immediately after the city of Orenburg, the first two villages attacked by the epidemic were the burgh of Rassipnoe and the village of Bicoulovoy, placed at a great distance from one another, upon two diverging routes, having intermediate to them a great number of other towns and villages which were not attacked at all or not until a later period, and also having much less communication with one another than each of them had with certain other places until then remaining unmolested by the disease."

In making this long extract, from a work to which we are indebted for many of the foregoing facts, we do not propose in this place, to enter on the difficult question of the mode in which Epidemic Cholera extends itself. We presume however, that some discussion of the circumstances of its entrance into Europe will not be without interest to our readers. Let us now inquire whether, in its extension over that continent, it has observed the same laws as in Asia.

However, it must be stated, that the first appearance of Epidemic Cholera in Europe, was in reality six years before, at Astrachan, in the delta of the river Volga, not far from the Caspian sea. But what is remarkable, after prevailing for a time, in that city, it ceased without extending beyond its limits.

In 1830 about the 1st of July, it reappeared, and soon began to show itself to the East, the North, and the West. In the second week of October it commenced in Moscow, where it prevailed till January. In the ensuing spring 1831, it broke out in Archangel on the white sea, in lat. 65 deg. and on the 12th of June commenced in St. Petersburg; where it raged for a time, ceased, and then recurred. A month before, it had manifested itself in Riga, at the mouth of the Dwina, in the S. W. corner of Russia proper; and at Danzig, another town on the Baltic sea, in Prussia; having already affected most of the vast country, between that sea and the Caspian. Mean while, extending along the shores of the sea of Azof and the northern side of the Black sea, it ravaged the southern provinces of Russia; and overspreading Hungary and Poland, extended beyond them into Prussia and Austria, quite to Hamburg, on the Elbe. Dipping down the Dardanelles, it affected Constantinople, and recrossing into Asia minor, fell with violence upon Smyrna, in lat. 38 degs. below which it did not occur.

As yet, it had not extended west of the 10th deg. of east longitude; but in the autumn of the same year, 1831, in which it ravaged the countries lying south of the Baltic, it appeared in England. Its onset was at Sunderland, a coal town, at the mouth of the river Wear, in the north eastern part of the Kingdom. Well authenticated cases occurred in August; but no alarm was sounded, till the month of October. It prevailed till February, extending to the towns and villages on the Wear and Tyne, including New Castle, Gateshead and other places, in the great coal district;—and, extending into Scotland, raged with considerable mortality, in the villages and country, quite to Edinburg and Glasgow inclusive. In the month of March, it appeared in London, where, however, it affected comparatively, but a small number. About the same time, without invading the centre and west of England, including the great commercial and manufacturing towns of Liverpool, Birmingham and Manchester, it opened on ill-fated Ireland, and over ran, most of the Island ; being attended in many places with great mortality. In May it broke out in Paris, and raged for two or three weeks, with deadly violence. What may have been its spread, in that kingdom, is not at this time known with any certainty. Thus, the parts of Europe which remain unvisited, are Holland and Belgium to the north, and the states which border on the Mediterranean sea—Greece, Italy, Naples, Switzerland, the southern part of France, Spain and Portugal, to the south.

Having briefly sketched the progress of the Epidemic in Europe, let us inquire, for a moment, whether its character has undergone a change, on that continent. Many of the facts necessary to this inquiry, are still wanting in this country. From what we have seen, we would say :—

1. That the laws of its propagation, seem to be the same in Europe, as in Asia. It has, as in its native country, extended itself along navigable rivers, and highways; sought out maritime towns; fallen heavily upon the great armies, engaged in advancing and resisting the plans of despotism; passed by villages and districts of country, affecting places on both sides, and beyond them; sought out the poor, the terrified, the afflicted, the badly lodged, clothed and fed ; the intemperate, the exposed, and the dwellers in damp and unventilated places. But it has, also, stricken many in the opposite of all these circumstances.

2. It has penetrated farther north in Europe than in Asia ; and, as yet, has spared, many southern countries, lying nearly in latitudes, which it has repeatedly ravaged in the latter.

3. It has prevailed more severely in the winter season, in the high latitudes of Europe, than in the mild climates of India and Persia.

4. In many instances, it has appeared to be communicated from man to man; while, as in Asia, the atmosphere of the sick has generally seemed to be harmless; and it has broken out in several places, where no suspicion of extraneous introduction could be raised.

5. Quarantines and military cordons, neglected, generally, in Asia, have been instituted and enforced in Europe, without resisting its progress.

6. The governments, and the medical men of Europe,—both contagionists and non-contagionists,—have satisfied themselves, that it cannot be propagated, by inanimate objects of any kind.

7. The character of the Epidemic in Europe, has varied a little in two respects from its Asiatic type : First, It has been much more commonly preceded by diarrhœa, or some kind of intestinal irritation; and secondly. much oftener followed, by a fever of the typhus form.

8. Its mortality, in proportion to the number attacked, has been greater in Europe than Asia. In the former it has sometimes been as high as one out of two, and frequently two out of five: while in Asia, the average mortality has not exceeded, one out of six or seven.

9. It is worthy of remark, that in London, a small number, proportionally, were affected, and the deaths exceedingly few, compared with many villages of the kingdom, allowing for the difference of population. In Paris, on the other hand, it atacked multitudes, ascended in many instances, into the higher ranks of society, and proved, extensively fatal; events well calculated to humble the pride of science; as that city is the great emporium of medical learning, the whole of which had been put into requisition, and such deliberate preparation made, for the reception of the pestilence, as led many of its physicians to predict, that it would prove but a small calamity to the people of France!

10. The cause or causes, which have produced such striking differences, in its mortality in different towns, have not yet been discovered.

Leaving the Old world, we come to contemplate Epidemic Cholera in the New.

From the gazettes it appears, that on the 8th of June of the present year, a malignant cholera morbus suddenly broke out at Quebec, among the Canadian French, and the wretched emigrants, chiefly from Ireland, who had during the spring been thrown in thousands upon the banks of the St. Lawrence. It quickly spread over the city, and affected many of the natives, in comfortable circumstances. In a few days it was at its height, and before two weeks had sensibly declined, indeed nearly ceased. On the 11th or 12th, the same malady manifested itself in Montreal, 120 miles up the St. Lawrence, where it raged with great mortality, but on the 24th of June, was nearly extinct. Meantime it made its appearance in many of the villages surrounding these two cities; and affected the Canadian French, still more, it is said, than the emigrants, multitudes of whom had ascended the river as far as Montreal and even above.

The question has been started, whether this be any thing else than the ordinary endemic of the country, in an aggravated form? It is impossible, from the data, which have, as yet, reached Cincinnati, to resolve this question; but from the suddenness of the invasion and its short duration, the multitudes taken down, its great prevalence among the poor and exposed, its alarming mortality, and the concourse of symptoms, we are compelled, till other facts shall be made public, to regard it as an extension of the Asiatic Epidemic. The question, whether it will extend into the United States, from Canada, will probably be answered in the affirmative, before these pages see the light. For whether propagated by contagion or atmospheric influence of any kind, its entrance into our country, is not likely to be prevented, unless the laws of its diffusion should be different in America from those of Europe and Asia. Moreover, we had last autumn, a wide spreading Influenza, so often the precurser of more violent Epidemics, and a visitant of Europe the year before the Cholera; and, at the present time, June 30, there is in this city, and no doubt, other parts of the United States, a great deal of diarrhœa, and many mild cases of endemic cholera morbus, both of which were harbingers of the Epidemic Cholera in England. The pestilence may not, however, overspread the United States during the present summer, for it has often lingered a year or more on the confines of a country without entering, and then made a sudden and mortal irruption.

A disease so wide spreading and fatal as Epidemic Cholera, must of course have a powerful and pervading remote cause, but what it is, has not yet been discovered. The ingenuity of the profession has suggested several, but strong objections lie against the whole. Let us review them.

### 1. Sol-Lunar Hypothesis.

Epidemic Cholera has been ascribed to the influence of sun and moon. But as the disease has been progressive through various latitudes and longitudes, and persons have been taken down at every hour, from the new, to the full moon, and from the latter to the former, the hypothesis of sol-lunar influence, would seem to be altogether visionary.

### 2. Cometary Influence.

It has been supposed to depend on the approach of a comet, which is presumed to exert an effect on the electricity of the earth or its atmosphere, unfavorable to human life. Such an influence, however, ought to bear some kind of astronomical relation to the great circles of the globe, and not to the courses of rivers, it should be simultaneous and universal, rather than local and meandering; moreover, the present Epidemic commenced at least fifteen years ago, and the comet now approaching our solar system, is not yet visible in the northern hemisphere, where the disease has chiefly prevailed;—facts that would seem to *disprove*, what is not sustained by any positive argument or phenomenon.

### 3. Geological Theory.

The malady has been attributed to exhalations from the bowels of the earth; and the occurrence of earthquakes in Persia, *since* it commenced, has been cited as evidence in support of this, which may be called the geological hypothesis. It has the dignity of having been cherished, if not first suggested, by Sydenham. It can scarcely be doubted that mineral fermentations, attended with the developement of various gasses, do actually take place beneath the surface of the earth; and that they sometimes escape into the atmosphere. But the progress of Epidemic Cholera bears a relation to the condition of the surface of the earth, rather than the strata which lie beneath; and it is difficult to conceive of the occurrence of exhalations, in the locomotive manner in which the epidemic has spread. On the whole, it must be admitted, that this doctrine is a mere hypothesis unsustained by any positive facts.

### 4. Miasmatic Theory.

Malaria, or poisonous air, formed by the decomposition of dead vegetable and animal matters, acted on by heat and moisture, has with confidence been cited as the remote cause. In support of this theory, it has been alledged, and with truth, that the disease prevails most, in hot and humid situations, which are supposed to send forth miasmatic exhalations; in close, dirty, and unventilated cities; and at the season of the year when intermittent and remittent fevers, *said* to depend on malaria, are prevalent. It has, moreover, not unfrequently put on, after the first paroxysm, the type of one of them, or some other fever. Thus, the miasmatic theory is far more conformable to the ascertained facts, than either of the others, just mentioned, and deserves grave consideration. When we recollect, however, that in several instances it has prevailed in dry places and at great elevations, that it has continued, in some cases, throughout the winter, and that it is, and always has been, absent from many places on the surface of the globe, where heat, moisture, and decomposable matter, all the requisites for the generation of malaria abound, we are unable to assent to the miasmatic hypothesis, although so plausibly supported.

### 5. Meteoratious Theory.

It has been held by many, to result from some change in the mode of union, or relative proportion of the gaseous, and etherial fluids, which constitute the atmosphere. To this supposed change, one of our able countrymen, Dr. Joseph Mather Smith, of the University of New York, has applied the term insensible *Meteoration.* He supposes, it to relate, chiefly, to the electric fluid; and, with many others, believes Epidemic Cholera to be the direct effect of some disturbance in the equilibrium of that fluid, or of electricity and magnetism combined. In the language of this school, cholera is an uncontagious, meteoratious epidemic, like influenza. Others have preferred the opinion, that the assumed meteoration involves some change in the gaseous constituents of the atmosphere, and is connected, in some manner, with an excess or deficiency of oxygen, azot, or carbonic acid. To make the theory, in any degree, adequate to the explanation of the phenomena, it is necessary to admit an influence, sufficient to begin and spread this meteoration progressively over the surface of the earth. The doctrine of ferments may be cited, in illustration of this supposed atmospheric change. A little leaven leaveneth the whole lump. A minute quantity of yeast, will start, in a large volume of fermentable liquid, an intestine movement, that will ultimately extend throughout the whole. Now may not a similar fermentative action be excited in the atmosphere, and extend successively over various places? Such a supposition would render the meteoratious hypothesis applicable to the geographical history of the Epidemic, and give to it at least a high degree of probability. It would account for the spread of the disease into diversified localities; for its occasional continuance through the winter; its progressive and sudden invasion, general prevalence in a particular district, and its sudden disappearance, after a brief reign of fifteen or twenty days. But the evidence of this meteoratious change, is wanting, or at least defective, and what is the ferment? Admitting the meteoration, we must still look further back for a cause to excite it. Such an agent may undoubtedly exist, but as yet the proofs of its reality are altogether conjectural.

### 6. Contagion.

Contagion has every where had its advocates. In Asia these are few and feeble. The natives, it is said, do not view the Epidemic as communicable from the sick to the well; and the majority of European physicians practising in that country, are opposed to the doctrine. The people of Europe, however, have dreaded it as contagious, the governments of Europe have met it as contagious, and the physicians of that continent have observed, experimented, disputed, wrangled, and written about its contagiousness, until they have split into several sects. A few believe it to be unconditionally and powerfully contagious, and capable of propagating itself without extraneous aid; a greater number hold, that it is contagious only in an impure or malarious atmosphere, from confined air, personal filth, or public sources of miasmata; others that a general meteoratious or atmospheric distemperature of some kind is a necessary co-operative agent; and others that is, under all circumstances, non-contagious. It would seem, that with the progres of the malady over Europe, the number of ultra contagionists has been constantly diminishing, and that of the non-contagionists increasing. This, to those who are at a distance from the theatre of observation, is a fact which is not without its value.

The naked question is, do the bodies of those who labor under Epidemic Cholera, secrete and throw out a poison, either liquid or ærial, which, inhaled or swallowed by a healthy person, will excite the *same* disease in him, and can he in the same manner, raise it in the system of a third? If so, the malady is contagious, and perpetuates itself by morbid secretion; otherwise it is not.

In support of the affirmative of this proposition, a great many facts are cited, going to show the communication of the disease from one person to another; the introduction of it into towns and villages by individuals affected with it; the immunity of families, small communities, and even cities, when they practised a rigid exclusion of all who were tainted; finally, the advocates of this theory dwell upon the advancement of the disease through fifty degrees of latitude, over a vast variety of soils, throughout all the seasons, and especially along high roads and navigable streams.

On the other hand, the non-contagionists refer to the acknowledged fact, that the medical and other attendants on the sick are but seldom attacked; that it frequently limits itself to a single member of a family; that it has not often *appeared* even, to spread from intercourse with the affected; that it has repeatedly shown itself in places, where it could not have been introduced by human agency; that it sometimes appears in all parts of a city, in a single night, and reaches thousands in a few days; that it has appeared simultaneously in the different parts of certain districts; that it has prevailed in one wing of an army without extending to the other; that a detachment of marching troops, laboring under the disease, has joined another encamped on a healthy spot, without communicating the malady; that it has broken out in ships long at sea, finally, that persons going from towns where it prevailed, have sickened in the country, and in villages, without infecting those around them.

To these positive facts, it may be added, first, that the measles, small pox, vaccine disease, itch, hydrophobia, and other maladies, which generally, or always, propagate themselves by the morbid secretions which they generate, are found to be operative on almost all persons, while, in many places, a very small number only are seized with cholera; secondly, that the same contagions are capable of raising in others the diseases of which they are respectively the products, independently of any predisposing or exciting causes; while the remote cause of Epidemic Cholera, whatever, it may be, is comparatively harmless, except in certain situations and under certain circumstances.

If then a contagion emanates from the bodies of cholera patients, it is much less active than that of small pox, can affect but a small number of those who live regularly, in comfortable circumstances; and is chiefly operative on those of enfeebled constitutions or bad habits, who live poorly, and are exposed, labor under apprehension of the disease, or breathe an atmosphere impure from other causes. Such a contagion *may* exist; but direct proofs are wanting, and we know of few analogies that could legitimately be brought to the support of the doctrine. A contagion which should require so great a variety of conditions to give it activity, would certainly not be very dreadful.

The subject of what has been termed contingent contagion properly falls into this place. The expression is not a correct one. A disease may be contingently communicable, but cannot be contingently contagious. A contagious effluvium, is either exhaled or not exhaled from the body of a person who is ill, according to the nature of the disease. In small pox, such an exhalation always occurs; in pleurisy, never. In mild cases of the former, the amount of exhalation may be so little, that under free ventilation, the disease would be much less communicable, than when the circumstances were different; but still it is *positively*, not *contingently* contagious. Let us apply this reasoning to Epidemic Cholera. In regard to contagiousness, it either belongs to the species which embraces small pox, or that which includes pleurisy. Suppose the former. If so, the Epidemic is *always* contagious, though the amount generated, may be so small, that unless the patient be kept in an unventilated place, where the contagious effluvia would accumulate, and those about him were strongly *predisposed*, by living in an atmosphere unhealthy from filth and defective ventilation, he could not communicate the disease to them. Now, that the Epidemic is contagious, even in this feeble and almost harmless degree, remains we think to be shown.

If the Epidemic Cholera be contagious, it is important to ascertain the greatest length of time that can elapse after an exposure, before the disease may show itself. Many contagionists have, occasionally witnessed the disease in a few hours, after they supposed the poison had been inhaled; two or three days have been stated as a more common period, and fourteen has, perhaps arbitrarily, been set forth, as the ultimate term, beyond which the virus could not lurk in the system without exciting the disease.

In the opinion of contagionists, can the clothing of a physician or friend, who visits the sick, communicate the disease to others? They have, we believe, unanimously decided this question in the negative. The same decision, we have already stated, has been made in regard to goods and inanimate objects of every kind. Actual exposure, then, to the atmosphere of the sick, is indispensable; but such exposure, it is admitted by all, will affect a very small number only.

### 7. Animalcular Hypothesis.

The last hypothesis which we shall mention, is the *animalcular.* To understand this, which is not a new doctrine, some acquaintance with the natural history of insects is indispensably necessary.

1. It is well known, that there are in fluids, and on many solid bodies, both living and dead, a great variety of insects too small to be seen by the naked eye. They have been revealed by the microscope. Derham in his Physico-Theology, London, 1727, says of *animalcules:*

"Most stagnated waters are stocked with them; new pits and ponds; yea, holes and gutters on the tops of houses and steeples.

It is almost impossible by reason of their perpetual motion and changing places, to count the number of animalcules, in only a drop of the green scum upon water; but I guess I have sometimes seen not fewer than one hundred frisking about in a drop no bigger than a pin's head. But in such a drop of pepper water, a far greater number, these being much less than those."

Baker, in his work on the microscope,* observes:

"It is common in summer-time, for the water that stands in ditches to appear sometimes of a greenish, and sometimes of a reddish color, which, upon examination with the microscope, is found entirely owing to infinite millions of *animalcules* crowded together on the surface of it, and giving it such appearance.

* London, 1764.

The water that drains from dunghills, and looks of a deep brown color, is so thronged with *animalcules*, that it seems to be all alive, and must be diluted with water before they can be sufficiently separated to distinguish their various kinds."

Finally, he remarks:

"The waters every where abound with life, and are an endless subject of employment for the microscope: seas, rivers, ponds, ditches, and almost every puddle, can, by its assistance present us with living wonders never before discovered."

2. By an admissible analogy, we may infer, that the air, equally with the waters, is peopled with these minute beings. We are, indeed, accustomed to see flying insects in vast multitudes, that are visible to the naked eye, only in a strong light and under certain aspects; and it is quite obvious, that flying animalcules *may* reside in the atmosphere, undiscoverable by magnifying glasses, as such instruments are not adapted to observations on the air.

3. Baker, in the work just quoted, has recorded a number of observations, which go to confirm this conclusion. The following extract, although rather long, will perhaps be read with interest, especially by those who know that the author was a man of science, and a distinguished member of the Royal Society.

"The smallest living creatures yet known, are the animalcules in fluids; whereof many kinds have been discovered by the microscope, of such exceeding minuteness, that a million of them would not equal the bigness of a large grain of sand; and it is probable there may be numberless species of a size much less than these. It is also likely that there are as many, or even more kinds of these *invisibles*, (if I may use the term,) than of those whose size is discernable by the naked eye. Here, therefore, is abundance of scope for inquiry and admiration; since every drop of water, or other liquor, (excepting oil and spirits,) either does already, or, upon standing exposed a few days, will appear full of *living creatures*, of various sizes and forms. Some kinds of these *animalcules* seem to be really fish, and are natural inhabitants of the water all their lives; others live there but occasionally, in the manner of gnats, which, from eggs dropped by their parents in the water, become swimming animals; but after a while, shed their skins, appear in a form that bears no resemblance to what they were before, take wing and turn creatures of the air.

We may thus account how water, wherein *pepper*, *hay*, *oats*, *wheat*, or other *vegetable substances* are infused, will soon become full of life; for those *minute* and *invisible little flies*, which are every where hovering in the air, and seeking places to deposite their eggs, when a fluid offers well stored with proper nourishment for their future offspring, may be supposed to resort to it in swarms, and lay their eggs there. These eggs being soon hatched, the infant brood swim about and live happily in the fluid; till, grown to their stated size, they in due time change their forms, employ their wings and fly away.

The truth of this I have often experienced; for after observing some kinds of *animalcules* in several fluids to be grown to a certain bigness, on a sudden, I have found them all gone away, and only a much smaller and consequently a younger race of the same kinds remaining; which, also, when grown to alike size, have soon after in the same manner been gone too. *Besides, if the infusion be covered, though with a muslin or fine lawn, I have constantly found that few animalcules have been produced therein; but upon taking off the cover, in a few days it will be full of life*; which seems to prove that the eggs, whence these *animalcules* come, must either be deposited by their own parents, as I above suppose, or be brought along with the air. And, indeed, both these ways may possibly be; for as the eggs of such minute creatures are lighter than air, millions of them may continually float therein, and, being wafted every where indifferently, may perish in places unsuitable to their nature, but hatch and thrive when they happen to be lodged in a proper *nidus* for them. Some people imagine that the eggs of these little creatures are lodged in the pepper, hay, or whatever else is put into the water; but, were it so, I cannot think a thin covering of lawn, which does not exclude the finer part of the air, would prevent their being hatched; and therefore must conclude it a mistake.

Though water that stands at rest, and exposed in the open air, will, after a few days, have some animalcules in it, they will be found in no degree, so numerous as when vegetable bodies have been steeped therein; for no creatures seem able to subsist on mere water only, and what little particles besides may accidentally happen in it can maintain no great number. But when, by infusion of the above mentioned substances, water is stored with their proper food, the microscope can show myriads of *living creatures* in every little drop."

It is not in warm weather only, that our ingenious philosopher found vegetable infusions to become impregnated with animalcules. The same happened in the 'midst of winter, if the water was not frozen,' but not so speedily.

4. The smallest flying insects which we observe by the naked eye, are gnats; these belong to the genus Culex of the entomologists. The *musquito*, in some systems is referred to the same family. Let us dwell for a moment on the character of the *Culices*. They deposit their eggs in stagnant or eddy water. A single gnat has been observed to discharge from 250 to 300;* these are hatched out, and the larvæ they produce swim for a time, and then acquiring wings, rise into the air. They, as well as the musquito, have an armed proboscis, by means of which they puncture the skin, and draw out the blood of the animals on which they subsist. The instinct of these minute insects, directs them, then, to wet places, for the purpose of depositing their eggs, and to man and animals, as affording them sustenance. The number of individuals belonging to a species is innumerable, and the generations rapidly succeed each other; they delight in a humid atmosphere, fly abroad chiefly in the evening, and can maintain themselves against a considerable breeze. Now, is it unreasonable to conjecture, that there are many of this tribe, invisible to the naked eye, that have the same instincts, habits, and modes of propagation with the visible species? And may they not deposite their eggs in vegetable infusions, such as were prepared and examined by Baker? It is a law of the animal kingdom, that the smaller the individuals of a species, the more numerous; we may conclude, then, that if there are invisible, ærial insects, the number of individuals may exceed all human computation.

5. Gnats and other small flying insects, whose bite is poisonous, abound both in hot and cold climates.

"One would at first imagine that regions where the polar winter extends its icy reign would not be much annoyed by insects; but however probable the supposition, it is the reverse of fact, for nowhere are gnats more numerous. These animals as well as the *tipulidæ*, seem endowed with the privilege of resisting any degree of cold, and of bearing any degree of heat. In Lapland their numbers are so prodigious as to be compared to a flight of snow when the flakes fall thickest, or to the dust of the earth. The natives cannot take a mouthful of food, or lie down to sleep in their cabins, unless they be fumigated almost to suffocation. In the air you cannot draw your breath without having your mouth and nostrils filled with them; and unguents of tar, fish-grease or cream, or nets steeped in fetid birch-oil, are scarcely sufficient to protect even the case-hardened cuticle of the Laplander from their bite. In certain districts of France, the accurate Reaumur informs us that he has seen people whose arms and legs have become quite monstrous from wounds inflicted by gnats; and in some cases in

*Kirby and Spence's Entomology.

such a state as to render it doubtful whether amputation would not be necessary. In the neighborhood of the Crimea, the Russian soldiers are obliged to sleep in sacks to defend themselves from the musquitos; and even this is not a sufficient security, for several of them die in consequence of mortification produced by the bites of these furious blood-suckers. This fact is related by Dr. Clarke, and to its probability his own painful experience enabled him to speak. He informs us that the bodies of himself and his companions, in spite of gloves, clothes, and handkerchiefs, were rendered one entire wound, and the consequent excessive irritation and swelling excited a considerable degree of fever. In a most sultry night, when not a breath of air was stirring, exhausted by fatigue, pain, and heat, he sought shelter in his carriage; and though almost suffocated, could not venture to open a window for fear of the musquitos. Swarms nevertheless found their way into his hiding place; and, in spite of the handkerchiefs with which he had bound up his head, filled his mouth, nostrils, and ears. In the midst of his torment he succeeded in lighting a lamp, which was extinguished in a moment by such a prodigious number of these insects, that their carcases actually filled the glass chimney, and formed a large conical heap over the burner. The noise they make in flying cannot be conceived by persons who have only heard gnats in England. It is to all that hear it a most fearful sound. Travellers and mariners who have visited warmer climates, give a similar account of the torments there inflicted by these little demons. One traveller in Africa complains that after a fifty miles' journey, they would not suffer him to rest, and that his face and hands appeared, from their bites, as if he was infected with the small-pox in its worst stage. In the East, at Batavia, Dr. Arnold, a most attentive and accurate observer, relates that their bite is the most venomous he ever felt, occasioning a most intolerable itching, which lasts several days. The sight or sound of a single one either prevented him from going to bed for a whole night, or obliged him to rise many times. The species which I have examined, is distinct from the common gnat, and appears to be nondescript.

From Humboldt also, we learn, that 'between the little harbor of Higuerote and the mouth of the Rio Unare the wretched inhabitants are accustomed to stretch themselves on the ground, and pass the night buried in the sand three or four inches deep, leaving out the head only, which they cover with a handkerchief.' This illustrious traveller has given an account in detail of these insect plagues, by which it appears that amongst them there are diurnal, crepuscular, and nocturnal species, or genera: *Musquitos* or *Simulia* flying in the day; the *Temporaneros*, probably a kind of *Culex*, flying during twilight; and the *Zancudos* or *Culices* in the night. So that there is no rest for the inhabitants from their torment day or night, except for a short interval between the retreat of one species and the attack of another. We learn from this author that the sting or bite of a *Simulium* is as bad as that of the *Stomoxys* before noticed.

It is not therefore incredible that Sapor, king of Persia, as is related, should have been compelled to raise the siege of Nisibis by a plague of gnats, which, attacking his elephants and beasts of burden, so caused the route of his army, whatever we may think of the miracle to which it was attributed; nor that the inhabitants of various cities, as Mouffet has collected from different authors, should, by any extraordinary multiplication of this plague, have been compelled to desert them; or, that by their power to do mischief, like other conquerors who have been the torment of the human race, they should have attained to fame, and have given their name to bays, towns, and even to considerable territories."*

Now may we not be permitted to conjecture, that species invisible to the naked eye, are also poisonous? The number of reptiles and insects, which secrete a venomous fluid, as a means of attack or defence, is really very great, and the disease produced by the venom of some of the former, as the rattlesnake, for example, bears a striking resemblance to Epidemic Cholera.

6. Certain *Culices*, as the musquito, extend themselves up and down rivers, from warmer to colder climates. Thus the insect just named, ascends the Mississippi and Ohio, as the hot weather comes on. May not microscopic species of the same family, follow the same law, and thus, at times, disseminate themselves into regions very remote from those to which they were indigenous?

7. But insects are dispersed over the earth in other modes. The winds may carry them when they do not make resistance, and even when they do, if the current be strong. Many insects, moreover, attach themselves peculiarly to man, especially when he lives in poverty and filth, or is enfeebled by disease; and may not this be the case with species that are invisible?

Let us apply these facts to the history of Epidemic Cholera.

We assume the existence of malaria, of mineral exhalations, of meteoration, of contagion; let us, in like manner, assume the existence of poisonous, invisible, ærial insects, of the same or similar habits with the gnat; let us assume still further, what will scarcely be denied, that these insects have instincts, which may direct their migrations, their search after food, and their choice of situations; and that they are liable to be carried by high winds into elevated and dry places, which they would not

* Kirby and Spence.

frequent from choice; finally, let us suppose that some cause has augmented indefinitely the number of individuals of some species, and we shall then have all the theory which is necessary to explain most of the facts connected with our Epidemic. This hypothesis would account for the greater prevalence of the disease in hot than cold countries, on the banks of rivers and near their mouths, over marshy grounds, about marine harbors, and in other humid situations, than under opposite circumstances, because there the enemy must multiply most. It also accounts for the connexion of the disease, with wet filth, and decomposing vegetable substances; as, in such matters, according to Reaumur and Baker, *animalcules* appear in countless multitudes. The doctrine likewise explains the simultaneous breaking out of the disease in various parts of the same country; its sudden invasion, short duration, and rapid decline. Its occasionally keeping on one side of a river, although the inhabitants of both perpetually cross and recross; its affecting one part of a town only; its extreme mortality in one wing of an army, while the other, encamped in a locality a little different, remained healthy; its appearance and disappearance from a body of marching troops, two or three times in succession, as they passed over different kinds of soil; its leaving many places untouched; its returning upon others, and ravaging them anew; its occasionally visiting dry, elevated, and salubrious situations, to which they might be carried by the winds or their own flight; and, perhaps, the fact of its not having crossed the arid deserts of Arabia into Africa. The doctrine still further explains, why exposure to a damp, and especially the evening air, has been found so productive of the disease—as the *animalculæ*, may, from analogy, be presumed to be then most abroad; why so many persons are attacked in the latter part of the night, particularly if exposed to the atmosphere of the evening; why a night breeze from the shore, in hot countries, blowing upon a vessel lying near, has sometimes been followed, in a few hours, by malignant cholera, in a great number of those exposed to it;* why persons residing in low, damp, filthy, and ill ventilated

* Johnson on Tropical Diseases.

places are more affected than others—for in such situations the insects would multiply rapidly, and no cause would operate to disperse them; why some persons are more liable to the disease than others—as we find the larger insects, more disposed to prey on some individuals than others; why persons enfeebled in constituti n are oftenest its victims—as other insects and vermin are constantly prone to select such as their prey; why masses of forest have sometimes seemed to be a protection—for many insects delight to gather about green trees; why the disease does not entirely cease, during winter, though it is generally mitigated; why it spreads, as the French Academy of Medicine expresses it, capriciously, that is, according to animal instinct; why it seemed to be so often imported when, in fact, it is not contagious—the animalcules might swarm and multiply in the hold of a vessel, and escaping when it reached the destined port, and depositing their *ova* in the water, might speedily reinforce themselves, and originate the malady; or during the voyage, might issue from beneath the decks, and infect the crew; why individuals going into healthy situations, and sickening with the disease, do not communicate it; why those who attend on patients where it is epidemic, are not oftener affected than others; why caravans have often appeared to introduce the disease into places—as the *animalcules* might adhere to, and travel with them, as is often the case with larger insects; finally, why quarantines are not to be relied on, and military cordons have proved entirely useless—as the enemy on his own or on the wings of the wind, might pass on unseen and unobstructed. It is in favor of this hypothesis that it calls for all the precautions that experience has shown to be necessary; the avoidance of every thing that produces debility; the removal of nuisances; the drying up of wet places; free ventilation; and seclusion from the evening and night air; to which end, the experiments of Baker, on the influence of gauze covers, in excluding *animalcules* from vessels containing vegetable infusions, would suggest for our windows, at night, the screens which Lancisi informs us have been found efficacious in preventing intermittents in Italy.

As to the manner in which the supposed ærial *animalculæ* might act on, or enter the human body, it may be the same as that in which contagion, or malaria—admitting its existence—exerts itself. The *ova* of such insects may even be deposited and float in the air, to fall into water and be hatched out, as the seeds of many of the more imperfect plants, such as the *fungus*, the *lycoperdon*, and *mucor*, are disseminated; and these *ova*, as well as the perfect insects, may be inhaled or swallowed, and adhere to the mucous membrane, or perhaps be absorbed into the blood, or both; or, the *ova*, being deposited in the waters, may be swallowed, and the insects developed in our bodies.

Such is the application that may be made of the animalcular doctrine to the history of the Epidemic Cholera. The greatest objection to this doctrine, is, that the Epidemic sometimes prevails in winter, when insects are generally torpid. We must grant the apparent force of this objection; but it would come with a bad grace from the advocates of malaria, as the same cold ought to put an end to miasmatic exhalations of every kind; while the general *abatement* of the disease in winter, is a fact adverse to the geological hypothesis, to the sol-lunar, the cometary, and, perhaps, the meteoratious. Moreover, many insects are considerably active in cold weather, and, finally, it should be recollected, that Mr. Baker found vegetable infusions, when exposed to the air in winter, provided they were not incrusted with ice, to become impregnated with animalcules, showing that these microscopic beings are really abroad when the weather is cold. They may in fact be so small as to float permanently in the atmosphere in a torpid condition.

We am, however, far from avowing belief in a reality of this theory, but disposed to think that it explains more of the facts, than either of the other hypothesis. This, however, does not establish its truth. A theory to be true, must explain *all* the facts, and be the only one that can. But, although the animalcular be a mere hypothesis, it may be useful to science to assert its claims. There are too many members of the profession, and of society at large, who allow themselves to adopt and dogmatically maintain some one of the other hypotheses which have been enumerated; and it may, possibly, excite doubts, and refresh the spirit of inquiry, for such philosophers to be shown, that a doctrine which they might have regarded as altogether fanciful, will, in reality, explain many points in the history of the Epidemic, quite as successfully as the systems, to which they yield an implicit assent.

Let us turn from the fruitless pursuits of a principal and universal remote cause of Epidemic Cholera, to the more profitable study of auxiliary causes. These, in theory, may be referred to two divisions; first, such as favor the generation or exalt the activity of the specific remote cause; and, secondly, such as predispose the body to its action, or quicken it into life. Every auxiliary cause must act in one of these two modes; but, after having established the classes, it may be difficult to assort the agents, which are to be distributed. Which of them, for example, should be referred to the first? None, we presume, but heat, moisture, decomposable matter, and confined air. These conditions are favorable to the generation of malaria and animalcular insects, and are calculated both to condense and disseminate contagion. Where they exist in the highest degree, the Epidemic, other circumstances being equal, has prevailed with the greatest mortality. It has, however, occurred in temperate weather, and in dry places, which were cleanly and well ventilated; and, therefore, it *has* a cause, distinct from these, or any, perhaps, that could be thus generated, the opinion of some writers to the contrary, notwithstanding. The circumstances which predispose the body to the action of that agent, whatever it may be, and are, therefore, referable to the second division, are exhaustion from age, chronic infirmities and innutricious diet; intemperance in the use of ardent spirits; long continued exertion; confined lodgings; the habitual breathing of an impure air; exposure to the damp and cool atmosphere of the night, after the intense heat of the day; irregular and excessive indulgence in food; apprehension of the disease, grief from the loss of friends; finally, constitutional temperament or native predisposition. The influence of these circumstances is so great, that those who are under several, or even one of them, are in great danger of being seized when the disease prevails; but it may occur in persons who are exempt from the operation of the whole, except the last, and there *is*, therefore, a cause distinct from them all, and their agency is limited to the effect of predisposing the system to its action.

If such a cause does not exist, why is the world now trembling at the geographical progress of an Epidemic, as uniform in its symptoms as small pox, and as fatal in its termination as the plague? The existence of such a cause, must, we think, be admitted. Whether it will ever be discovered is extremely doubtful. Meanwhile, philanthropy and science should exert themselves in correcting, or removing all the conditions that co-operate with it in the work of human destruction, and thus disarm, it they cannot slay the monster.

### TREATMENT.

As a suplement to this historical and ætiological view of Epidemic Cholera, we propose to say something on its treatment. in addition to the extended article in our last number. That article was, chiefly, made up of an account of the methods of cure pursued in Asia. We were, at that time, nearly ignorant of the results ot European experience. Since it went to press. a variety of information down to April last, has reached us—a part in the form of separate narratives and treatises. but more, in the celebrated Medico-Chirurgical Review, of which we shall avail ourselves as far as our limits will permit.

No man living has, perhaps, given equal attention to the history of Epidemic Cholera, with the able and indefatigable Editor of that Review. His interesting and valuable work on the diseases of hot climates, presents us with some of the earliest and best accounts of the Epidemic, or rather with some of the best suggestions for its treatment, especially by bloodletting and calomel. But it is in the latter numbers of his review, that we meet with the most copious streams of information aggregated from every quarter. At a meeting of the *Westminster Medical Society*, November 20, 1831, the Editor, Dr. Johnson, submitted in reference to this disease, a series of propositions, which we propose to extract from the number for January 1832.

PROPOSITIONS.

"I. That in *epidemic* cholera, as in most other epidemics, a poison or se[illegible] principle, whether emanating from the earth, from animal or vegetable bodies on the earth, or engendered in the air, strikes a predisposed individual, and, after an uncertain period of incubation, produces a train of phenomena, forming the subject of subsequent propositions. In *sporadic* cholera, the general or diffusive cause is absent; but when the common exciting causes are strong, and the subject highly predisposed, severe or fatal cases will occur, where the *symptoms* cannot be distinguished from those of the epidemic cholera.

II The effects of the choleric poison exhibit a great analogy to those produced by the virulent contagion of typhus, and the concentrated miasmal exhalations that give rise to malignant fevers, remittent and unremittent; such as have been seen in Batavia and other highly malarious places.

III. This poison shows its effects according to the evidence of our senses, *first*, on the nervous system, as evinced bythe prostration of strength,—by the affection of the head,—by the arrest of the secretions (dependent on nervous energy,) and, in fact, by a depression of the whole of the sensorial functions, as well as those of the organic life.

IV. The *secondary* effects of the choleric poison are shown in the vascular system The heart acts feebly,—the circulation recedes from the surface, and the blood accumulates in the vessels of the internal organs; decarbonization and calorification cease, or are greatly diminished; the temperature of the body falls to that of surrounding inanimate substances; paleness is changed to blueness; and the influence of the ganglionic system of nerves seems to be nearly suspended, if not annihilated.

V. It is at this period that nature appears to make violent, but too often unsuccessful efforts, to restore the broken balance of the circulation and to re-establish the secretions, by sickness and purging; the ejected fluids being exudations rather than secretions.

VI. If nature does not succeed by the above-mentioned efforts to restore circulation, secretion, and consequently oxygenation and calorification, these efforts themselves prove auxiliaries to the choleric poison in destroying life.

VII. We are not, in our present state of knowledge, certain whether the spasms be merely the effects of the poison on the nervous system, or an effort of nature to resist it; but they, like the sickness and purging, tend ultimately to exhaust the powers of life.

VIII. If nature, (by which I mean the constitution,)whether with or without aid, be able to resist the first or depressive shock of the poison, and institute a reaction in the system, that reaction, in a great majority of cases, becomes a fever, exhibiting a new train of phenomena, and demanding a different mode of treatment. If this view be correct it would lead to the inference that the choleric symptoms constitute the first or cold stage of a choleric fever.

IX. If reaction, with restoration of circulation, secretion, and oxygenation, do not take place, the patient dies in a state of asphyxia, the intellectual powers often remaining but little impaired, till the last glimmer of the lamp of life is extinguished. This has been often witnessed in concentrated miasmal fevers, both within and without the tropics.

X. Pathology.—All the changes which present themselves in the dead body are, in my opinion, *effects*, not causes, of the disease; with the exception of the congestion of black blood in the internal organs, which is almost the only phenomenon observable when cholera terminates fatally in a few hours. The traces of inflammation in various organs after death, indicate the causes or effects of the reactive fever, rather than of the cholera which precedes that fever.

XI. Treatment.—As we have no means of expelling or neutralising the poison, we can only endeavour to counteract its effects, and to assist nature in her remedial movements.

XII. The primary or essential indication is to restore the equilibrium of the circulation. That equilibrium effected, a restoration of secretion, calorification, and oxygenation follows.

XIII. The balance of the circulation is to be restored partly by internal, partly by external means; but always by several means simultaneously employed at the very earliest period of the disease.

XIV. Venesection may appear a desperate remedy, but we have a desperate disease to combat. I proposed this measure many years before the epidemic broke out, and it has been adopted to a very considerable extent, both in Asia and Europe. I proposed, and would still propose, venesection with a two-fold view; first, to relieve the heart and internal organs from a portion of that deluge of black blood in which they may be said to be drowning;—secondly, to turn, as it were, the tide of the circulation from the centre to the surface of the body. This measure I would chiefly confine to the young, the robust, and the previously healthy, and in them contemporaneously with, or subsequently to, the measure which forms my next proposition.

XV. The first internal remedy which I propose, both in aid and in imitation of nature, is a stimulant emetic, as infusion of mustard seed, or what perhaps would be better, the sulphate of zinc. I propose this from a conviction founded on observation, that of all the means which nature or art can bring into operation, the act of *full vomiting* is the most powerful in driving the blood from the trunks to the capillaries—from the internal organs to the periphery of the body. It is also the most universal excitant of secretion in every glandular structure of the living machine. Nausea and retching are quite different in their effects from the operation of full vomiting. Nausea and retching depress the power of the heart and nervous system, and prevent the blood from flowing to the surface; full vomiting impels the circulation with such force into the superficial vessels, that it is extremely difficult to stop the flow of blood from the orifice of a vein during vomiting. I have seen the blood come from a vein under such circumstances, with all the characters, or at least the appearance, of arterial blood. This proposition is well exemplified by sea-sickness, of which I have had painful personal experience. An unfortunate landsman bears a close resemblance, during the first storm at sea, to a man with cholera. He staggers about the deck, or clings to the railings of the lee-gangway, striving to keep down the rebellious heavings of his own stomach. But all wont do; up it comes; and during the first vomitings I have seen the blood gush from mouth, nose, and even the eyes of the sea-sick sufferer. From being actually blue with nausea, his face becomes red with vomiting. But the cause of the sickness still continuing, he ultimately becomes pale and exhausted ; he is like a man who takes a fresh dose of tartar emetic, after each paroxysm of vomiting. The wonder is that he does not die; some *have* died. It is curious that Cœlius Aurelianus, who gives the best ancient account of cholera morbus, places sea-voyaging among the causes of that disease.

It is but justice to state that Mr. Boyle proposed and practised *full vomiting* in the epidemic cholera ten years ago; and I have the very best authority for affirming that this practice, when it was pursued on the Continent, was eminently beneficial.

XVI. As soon as vomiting has produced its salutary effect on the circulation, or has failed to produce that effect, after a fair trial, I would propose diffusible stimuli with calomel and opiates, but not in immoderate doses. Brandy and laudanum, as the most readily procured, and the least likely to be loathed, are perhaps the best. But the choice of stimulants must be left to the practitioner; and the danger of inducing subsequent inflammation should be carefully borne in mind. Calomel alone would probably be the best medicine after the emetic.

XVII. The remedies above-mentioned, in moderate but efficient doses, seem to impart vigour to the heart and nervous system, through the medium of the stomach, while they mitigate the spasms, and restrain the useless or injurious exudations of fluids from the intestinal canal, the mercury changing that exudation into secretion.

N. B. Would not the inhalation of oxygen gas be beneficial?

XVIII. The external remedies are three—heat, friction and counter-irritation. These three means should be employed, not only simultaneously with respect to each other, but contemporaneously with the internal remedies. They should also be so employed, that the patient may not be required to throw a single voluntary muscle into action. Every muscular movement, even that of sitting up in bed, is prejudicial, or absolutely dangerous, during the exhausting orgasm of cholera. An apparatus permitting the application of heat, friction, and counter-irritation, without the necessity for any muscular exertion on the part of the patient, will be shown to the Society.

Query—As the cramps and spasms are chiefly confined to the extremities, and as the exhausting pain of these cramps is in proportion to the contraction and swelling of the affected muscles, would not firm compression by a bandage mitigate the spasms? Patients in cholera often cry out for the most violent extension and pressure of the cramped muscles.

XIX. Prophylaxis.—Conceiving myself, as well as every member of this Society, bound to abstain from all discussion of the question of contagion, I shall condense the subject of prevention into four words,—temperance, cleanliness, ventilation, and fearlessness:—in fine, the pursuance of all those means which tend to preserve general health, and the avoiding of all those causes which predispose to the common, or indigenous diseases of our own climate."

On the whole, the treatment of the cholera in Europe has not differed widely from that of Asia, but venesection and calomel have been less depended on in the former than the latter country.

### Bloodletting.

Mr. Greenhow of New Castle, whose book is one of the best we have seen on the Epidemic as it appeared in the north of England, speaks of this remedy in the following terms:—

"I have hitherto said little on the subject of this powerful remedy—powerful when employed at the auspicious moment—powerless, when attempted to be used at a later or an earlier period. When we are fortunate enough to be called to a patient before the pulse fails, still more before the serous evacuations commence, when he is suffering from the symptoms which so frequently occur in the first stage—nausea or vomiting, purging of billious matter, vertigo, head-ache, probably injested conjunctivæ, pain in the abdomen or at the pit of the stomach, with a quick, sharp, or oppressed pulse, and probably occasional cramps in the legs—a full bleeding will be found of the greatest benefit, not only in relieving the existing symptoms, but in averting the impending horrors of the second stage of the disease; this effect may perhaps yet be produced, although the pulse have become feeble and still more oppressed, but not when imperceptible. In such cases it expands and increases in strength and freedom, as the blood flows. If, however, asphyxia, coldness, and blueness of the extremities have fairly established themselves, the attempt to obtain blood is vain; thickened and stagnant as it is in the vessels, it cannot be made to flow, and if a few ounces be squeezed from the orifices, it hangs from them in long strings, accumulating like stalactites, without producing any beneficial effects. On the contrary, it fatigues the patient, exposes him to the prejudicial influence of cold, and suspends for a time more efficient means of relief. I must, therefore, hold bleeding in these circumstances to be inadmissible, principally, because it cannot be accomplished; and the attempt injurious, since it directs attention from measures of less doubtful utility because they are really practicable."

It is a *desideratum*, to know by the symptoms, which cases will admit and which not admit of blood-letting. Laennec lays it down as a rule of practice in all diseases, that if the pulse should be weak, but the impulse of the heart strong, we may bleed, but if both are weak, venesection is prohibited. Perhaps this rule might be applied to Cholera, and thus the stethoscope be made to guide us through the difficulty. In the absence of the means of making an *a priori* decision on this point, it will only remain to observe the effects of the flow of blood, and terminate it when the circulation becomes more feeble and limited, instead of expanding.

### Tartar Emetic.

Dr. Reich, of Berlin, in a pamphlet on Cholera, appears as the advocate of tartar emetic.

"His plan of treatment was this. Dissolve ten grains of emetic tartar in seven ounces of water, and give half an ounce every two hours, continuing the medicine, even after the evacuations have ceased, till appetite and natural fœces are restored. The first effects are, more copious purging than vomiting; but these soon cease, *tolerance* of medicine being, in a short time, established. He allows the patient plenty of cold drink—prohibits all heat and frictions—gives no other internal remedy—and states that, by the above measure, he cured 119 out of 123 patients."*

### Cathartics.

The Med. Chir. Rev. strongly recommends the following formula in the premonitory diarrhœa:

Rhubarb, half a drachm,
Ginger, five grains,
Brandy, one ounce,
Water, the same—mix.

When the bowels have been already well evacuated, this mixture may be infused and strained, and two ounces of water, with 20 grains of soda or magnesia added to it.

Dr. Dodd, of the same country, has seen croton oil beneficial in the stage of collapse. According to that gentleman, 'it abates the spasms and restores the secretions.'

### Injections of warm water.

In our last number we asked the question, whether drinking large draughts of hot water might not be beneficial? A modification of this practice has been adopted in England. Mr. Greenhow, has thrown into the bowels, by a forcing syringe, three or four pints of water, *as hot as it could be borne*, rendered stimulating with brandy, camphorated spirit, or Castile soap,

*Medico-Chirurgical Review, April 1832.

and speaks in high terms of its efficacy. The water appeared to communicate its heat to the neighbouring organs. Mr. Fife pursued the same method, sometimes adding laudanum to the water, with a similar result. He observed that when the water was discharged, it was 'astonishingly reduced in temperature.' This practice seems to us to promise a great deal. When the discharges have been very great, would not an infusion of nut-gall act beneficially on the mucous membrane of the bowels?

### Tobacco Injections.

An infusion of tobacco administered as an injection has been proposed by Mr. Baird, and employed by himself and some other gentlemen of New Castle, Eng. He has reported several cases, in which this medicine proved beneficial; and avers that he has not seen it do harm in any. He was led to its employment by the conviction, that the prostration of the circulation arose chiefly from a prolonged contraction, or systolic state of the ventricles of the heart, which he proposed to relax by the impress of tobacco.

"The first change,' says he, 'which takes place after the exhibition of the injection, is restoration of the circulation, as evinced by the increase of volume in the pulse, and restoration of the livid parts of the body to a more healthy hue. The cessation of cramps next ensues, and afterwards the suspension of vomiting and purging. Last of all, the re-establishment of the bilious and urinary secretions."*

The infusion should not be so strong as that directed in the books for strangulated hernia.

We shall leave the reader to form his own conclusions relative to this medicine. It certainly should not be ventured on, except in vigorous, masculine constitutions, and in the early stage of the disease. If used at all, it should perhaps be preferred when the spasms are violent.

### Morphia.

We do not discover that the salts of morphia have as yet received a sufficient trial, to test their efficacy in comparison with opium. The following formula would perhaps prove useful.

* Mr. Greenhow.

R. Sulphate of morphia, two grains;
Sulphuric ether, two drachms;
Simple syrup, four ounces, Mix.
A table spoonful for a dose, in some water.

LYE OF WOODASHES.

One of the best antacids in common cholera morbus, is a weak lye of fresh ashes and soot. It deserves a trial in Epidemic Cholera. It may be prepared in a few minutes, and the patient should frequently drink a wine glass full of it.

It is, probably, in the early period of the disease, that acidity prevails in the stomach and bowels, and antacids are called for. In more advanced stages, when the serous portions of the blood begin to exude into the bowels, the carbonate of soda of that fluid predominates, in the latter, when, of course, antacids are not required.

LIQUID AMMONIA.

In the report of the French Academicians we find the following:—

"M. Noel, in the midst of a pretty extensive epidemic, performed wonders by means of liquid ammonia, given several times a day in an aromatic infusion. M. Deville quickly dissipated the disturbances by the aid of a large dose of ether, administered immediately on the accession of the disease. Every where opium has been associated with diffusibles, with musk, with camphor, ether, or essence of mint; every where have been prescribed with success, tonics, bitters, and aromatics."

SUBNITRATE OF BISMUTH.

The same report says:—

"Quite recently, Doctor Leo has endeavoured to prove, from a great number of convincing facts, that the method most constantly efficacious against the Cholera, consists in the employment of sufficient doses of the sub-nitrate of Bismuth, one of the most active antispasmodics with which we are acquainted."

In protracted stages of collapse, it should be recollected, that the stimulants and antispasmodics must be frequently changed. The system soon loses its susceptibility to any one of them, but may still be excitable to another.

SULPHUR BATH.

Would not the sulphur bath prove beneficial in this stage of the disease, by relaxing the spasms, as well as communicating heat? Sulphur once had considerable reputation as an anti-spasmodic. If there was any foundation for this pretension in its favor, one might think well of trying it in Cholera. Taking a sulphur bath is generally followed by copious perspiration.

### Cold Affusion.

This is a Persian remedy. It has been adopted with advantage in Europe. Dr. Ainsworth, one of the Sunderland practitioners, after speaking of the inefficiency of certain remedies, observes:

"It is quite evident, then, that another line of practice must be adopted, and this should be the use of cold affusion; a practice much in vogue in the East, and since introduced into the Cholera hospitals of the continent, with marked success.

'One of the physicians of the Cholera hospital at Berlin, in writing upon the subject, says: " In those living corpses which are struck with asphyxia, lying cold, and without any pulse, external or internal stimuli cease to be so; inasmuch as the debilitated asphyxiated frame cannot, in its turn, act upon them. No steam apparatus, however vaunted; no warm bathing; no friction; no excitement, is sufficient in these cases." And this is what I am sure every person who has seen the disease, will coincide in. Though producd from internally, outwards, and not externally acting inwards, *asphyxia pestilentia* bears a strong relation to death by frost, in which there is an icy coldness of the surface, a want of pulse, and great congestion of the central parts. In these cases, we use frictions of cold snow, &c. until a gradual warmth is restored; and it is on the same principle, that sudden cold affusions, are indicated in Cholera. So forcibly did this strike medical men in this country, as a neglected remedial measure, that when the *Berlin Cholera Gazette*, which contained the notice of its successful employment, was made known, every writer was anxious to show that he had himself previously advocated its adoption.

'The patient is placed in an empty and dry bathing vessel or tub, and several buckets of cold water are poured on him, while the regions of the stomach and back are subjected to a kind of shampooing, or friction; and this process must be repeated, if the urgency of the circumstances requires it. No physic is given, and cold water is allowed for beverage. If the pulse revives, the affusions are continued in a tepid bath, and the patient is put to bed, where perspiration is excited by gentle frictions with cold flannels. It must be kept carefully in mind, that cold affusions are only applicable to the second period of the disease, and not to the first; and it is not a universal remedy, but can only be used in particular cases. To secure the convalescence of the patient, it is only necessary that he should be carefully watched, and all symptoms of returning heat and vitality, or recurrence of the usual secretions, be assisted by the exhibition of warm restoratives and gentle aperients, taking care to avoid local inflammation."

We are disposed to view this practice with great approbation. The water should be very cold, dashed on suddenly, and the application not continued very long. The *shock* imparted in this manner to the nervous system, could scarcely fail to be reviving. The subsequent hot applications, frictions and irritants, would have greater effect. The hot and cold bath might, indeed, be employed alternately. We should expect much from such a practice.

### Injections into the Veins.

In the Med. Chir. Rev. there is an account of experiments by Drs. O'Shaughnessy and Clanny, on the state of the blood in Epidemic Cholera, from which it appears that the serum of the blood drains off, or at least, that its saline ingredients chiefly escape, through the mucous membrane of the alimentary tube. This discovery has suggested to Dr. Latta, of Eng. the injection into the veins, of a solution, designed to supply the place of these salts, essential to the constitution of the blood. We are indebted to the non-professional journals for the following account, which at the present crisis seems worthy of being transferred to our pages. The quotations marked are said to be from the pen of Dr. Latta himself, who says:—

"The first subject of experiment was an aged female, on whom all the usual remedies had been fully tried, without producing one good symptom; the disease, uninterrupted, holding steadily on its course. She had apparently reached the last moments of her earthly existence, and now nothing could injure her—indeed, so entirely was she reduced, that I feared I should be unable to get my apparatus ready ere she expired. Having inserted a tube into the basilic vein, cautiously—anxiously I watched the effects: ounce after ounce was injected, but no visible change was produced. Still persevering, I thought she began to breathe less laboriously—soon the sharpened features and sunken eye, and fallen jaw, pale and cold, bearing the manifest impress of death's signet, began to glow with returning animation; the pulse which had long ceased, returned to the wrist; at first small and quick, by degrees it became more and more distinct, fuller, slower, and firmer, and in the short space of half an hour, when six pints had been injected, she expressed in a firm voice that she was free from all uneasiness, actually became jocular and fancied all she needed was a a little sleep. Her extremities were warm, and every feature bore the aspect of comfort and health. This being my first case I fancied my patient secure, and from my great need of a little repose, left her in charge of the hospital surgeon; but I had not been long gone, ere the vomiting and purging recurring, soon reduced her to her former state of debility. I was not apprised of the event, and she sunk in five and a half hours after I left her. As she had previously been of a sound constitution, I have no doubt that the case would have issued in complete reaction, had the remedy which already had produced such effect, been repeated.

Not having by me the number of the *Lancet* containing Dr. O'Shaughnessy's analysis, I adopted that of Dr. Marcet, only allowing a smaller proportion of saline ingredients. This I now find to be considerably less than natural, according to the more recent analysis I dissolved from two to three drachms of muriate of soda and two scruples of the subcarbonate of soda in six pints of water, and injected it at temperature 112 deg. Fah. If the temperature is so low as a hundred, it produces an extreme sense of cold, with rigors, and if it reaches 115 deg. it suddenly excites the heart, the countenance becomes flushed, and the patient complains of great weakness. At first there is but little felt by the patient, and symptoms continue unaltered, until the blood, mingled with the injected liquid, becomes warm and fluid; the improvement in the pulse and countenance is almost simultaneous, the cadaverous expression gradually gives place to appearances of returning animation, the horrid oppression at the præcordia goes off, the sunken turn-up eye, half covered by the palpebræ, becomes gradually fuller, till it sparkles with the brilliancy of health, the livid hue disappears, the warmth of body returns, and it regains its natural color,—words are no more uttered in whispers, the voice first acquires its true cholera tone, and ultimately its wonted energy, and the poor patient, who but a few minutes before was oppressed with sickness, vomiting, and burning thirst, is suddenly relieved from every distressing symptom; blood now drawn, exhibits on exposure to air its natural florid hue.

Such symptoms, so gratifying both to the sick and the physician, must never allow the latter to relax in his care—the utmost vigilance is still necessary. At first the change is so great that he may fancy all is accomplished, and leave his post for awhile. The diarrhœa occurring, he may find his patient, after the lapse of two or three hours, as low as ever. As soon as reaction by the first injection is produced, mild warm stimulants, such as weak gin-toddy mixed with some astringent, should be freely and assiduously administered. An attempt should be made to fill the colon with some astringent fluid. That such is requisite, is evident from the watery diarrhœa returning with violence, and if not restrained, death will ultimately make sure of his victim; therefore, so soon as the pulse falls, and the features again shrink, the venous injection must be repeated, taking care that the fluid in use retains its proper temperature. The injection should be carried on very slowly, unless the patient is much exhausted, when it may be used more rapidly at first, until a little excitement is produced, after which it should not exceed two or three ounces per minute, and now is the time for the exhibition of astringents by the mouth, which will be retained, for in general the sickness entirely leaves during the operation.

Such remedies must be persisted in, and repeated as symptoms demand, or until reaction is permanently established. I have witnessed no violent symptoms accompanying the rapid injection of the fluid, but I have thought that the hasty repletion of the system was followed by great increase of the evacuations, and consequently a more sudden depression of the powers of life. The quantity to be injected depends upon the effect produced, and the repetition on the demands of the system, which generally vary according to the violence of the diarrhœa; the greater the degree of collapse, the greater will be the quantity needed, though not uniformly, for a very slight loss produces much depression in some systems; hence there is often great collapse without much vomiting, purging, or cutaneous discharge.

Although in every case, even the most desperate, the cholera symptoms were removed, some of the cases failed, which I attributed to one or other of the following causes; either the quantity injected was too small, or its effects were rendered abortive by extensive organic disease, or its application was too late.

I have already given an instance where deficiency in quantity was the cause of failure, which I will now contrast with one in which it was used freely. A female aged 50, very destitute, but previously in good health, was on the 15th instant, at four A. M. seized with cholera in its most violent form, and by half past nine was reduced to a most hopeless state. The pulse was quite gone, even in the axilla, and strength so much exhausted that I had resolved not to try the effects of the injection, conceiving the poor woman's case to be hopeless, and that the failure of the experiment might afford the prejudiced and illiberal an opportunity to stigmatise the practice; however, I at length thought I would give her a chance, and in the presence of Drs. Lewins and Cragie, and Messrs. Sibson and Peterson, I injected one hundred and twenty ounces, when like the effects of magic, instead of the palid aspect of one whom death had sealed as his own, the vital tide was restored: but diarrhœa recurred, and in three hours again she sunk, One hundred and twenty ounces more were injected again with the same good effect. In this case three hundred and twenty ounces were so used in 12 hours, when reaction was completely reestablished; and in 48 hours she smoked her pipe free from distemper. She was then, for better accommodation, carried to the hospital, where probably from contagion, slight typhoid symptoms were produced. She is now, however, convalescent."

The Lancet contains the following summary of experiments, but we strongly recommend to the perusal of our medical readers the whole of the communications of Drs. Latta, Lewins and Cragie, which will be found in the above mentioned periodical. The editor of the Lancet, after giving the correspondence says—

"From the conjoint testimony of the highly respectable individuals who have conducted the various cases, it appears certain that of fifteen hopeless and abandoned cases, five were rescued from apparently certain death, by the treatment adopted, while of those which proved fatal, all but one had been complicated with such extensive organic disease, that no method of medication could do more than postpone a little the fatal event.—That reprieve the injection seems to have procured. In short according to the evidence before us, the method has only failed in one case in which it has been fairly tried—that is, where no organic disease had pre-existed, and where enough of life was left to sanction the least anticipation of success.,,

### Treatment of the first stage, in Paris.

But little relative to the treatment of Cholera in Paris has yet reached this country. The newspapers furnish us with the following, translated from a French gazette. As the recommendation seems, *prima facie*, to be judicious, we shall transcribe it entire. The author, it is stated, is one of the physicians of the *Hotel Dieu*. The recommendations, it will be observed, are for the first or premonitory stage.

"Notwithstanding the repugnance I feel to draw public attention to my own professional career, I cannot resist the desire I have to make known the invariable success of a preservative method which is no less simple than it is easy to put in execution. The imperious call of humanity and the general inquietude impose this on me as a duty. I dare therefore hope that my intention will not be misunderstood.

Cholera, amongst persons attended to at their own houses, has always been marked by precursory phenomena, which may be considered its first stage. The serious symptoms have always been preceded either by a slight pain in the stomach or by dull pains in the intestines, with looseness of the bowels, very often even by looseness of the bowels, without any pain whatever.

Sometimes these trifling symptoms, which hardly excite attention, are accompanied by an oppressive sensation, of which the sick state the seat to be in the pit of the stomach, (epigastric region.) Headache and at times vertigo have also been observed, also a numbness and twitching in the extremities, and a tendency to chilliness in some parts of the body.

At this stage the disorder is always curable, and the success of the following mode of treatment has never been doubtful. If then one or more of these symptoms are perceptible, the following course ought to be immediately followed:

1. Keep your bed constantly during four hours at least, after having added to it one or two additional coverings.
2. Apply immediately to the epigastric region 20 or 40 leeches according to the severity of the case.
3. Keep on the belly emollient cataplasms, always warm.

4. Drink copiously of a slight infusion of linden or mallows, cammomile, or balm, warm.

5. Observe a rigorous diet.

If by the employ of these means, an abundant perspiration is produced and lasts for some days, the dangerous stages of the disease are prevented, provided recourse has been had to these means from its commencement. It must not be lost sight of, that when an epidemic reigns, all indispositions remain subject to the influence which produced them, and may degenerate into the serious stages.

I think also that a good effect will be produced by persons in the enjoyment of health, undergoing this prophylactic regimen. It cannot be in any case injurious, though it may be unnecessary. I will guarantee that by this simple method, so easily followed, the scourge may be neutralized which is desolating the capital and throwing alarm into our provinces. This assertion is made on the strength of my own experience and on the success of a mode of treatment which has never in any one instance failed." BALLY.

### Treatment of the Broussaists.

We have been favored by a friend, who has just received it, with a pamphlet composed of two clinical lectures on the Epidemic, delivered in Paris, by Dr. Broussais. We note in them nothing worthy of attention, but the treatment. The distinguished author informs us, that he shall, for the sake of perspicuity, divide the methods of cure which have been pursued in the French metropolis in the following manner: "The *Ancient treatment;* or treatment of the sporadic cholera—*Treatment after the manner of Brown*, (stimulating)——*Mitigated treatment*, or eclectic or vaccillating treatment—finally, *Physiological treatment*, such as we employ here."

After briefly considering and condemning each of the three former, our learned author proceeds:—

"We come now to the *physiological treatment*, which we employ. I wish to describe, and if possible, justify it. We made, at first, some experiments with hot drinks and stimulants, moved, as we were, by the coldness of the patients: but as these means did not succeed, we abandoned them, and have not recurred to them. I watched the patients carefully; I gave them, not cammomile, for I dared not go so far, but mallows, and similar things. They said to me, 'I beg you to give me something cold; I am tortured by the hot drinks; I have a terrible burning in the throat, and I beg you to cool it in some way.' Their countenance brightened in saying this, but they afterwards fell into a still greater dejection. I concluded, from examining the bodies, and from the confession also of the patients, that stimulants were of no use. I then administered cold drinks; the patients drank freely, but the evacuations redoubled in consequence. I recollected that ice had been employed to advantage in Germany; but I only had a vague and unsatisfactory idea of the manner in which it had been used. It occurred to me to diminish the drinks and to give them ice. When the patients had copious evacuations both ways, I caused ice to be given them to eat, with the injunction to swallow it. They took the ice with delight: the tongue was cold, the pulse extinct, and the body chilled. When the tongue is observed to become red, the skin to resume its color, and the blackness to disappear, the ice may be discontinued and the drinks given; but while the physician is endeavoring to restore the moisture of the mouth and the interior of the body, the gastrite is developed, reaction is going on, the nature of the inflammation is changed, and consists in a rapid congestion towards the digestive canal. The vomitings and stools continue, and the pulse is accelerated; from being slight and hard, it becomes fuller and softer, the brown color of the skin passes gradually away, and one is surprised the next day, to see the patient with the signs of commencing inflammation of the stomach and bowels. In the mean time, when he is troubled with thirst, you may give him some drinks, which you may be sure he will absorb. The danger is of filling the intestinal canal at the moment it is choaked up. When the asphyxia and blackness have disappeared, and the patient has recovered his strength, you conduct him slowly without stimulus, waiting till he has become slightly cold, and till the tongue, which had become a pale red, but not more pale than when it first grew cold, has recovered its ordinary color. This is the substance of the treatment for the interior."

To the exterior, hot applications may be made, especially to the lower extremities. The breast should be left uncovered. Frictions should be prohibited. Leeches to the epigastrium are strongly recommended: bloodletting from the arm, except at the commencement of the disease, is forbidden. Diffusible stimulants of every kind—even Siedlitz water, he thinks pernicious. Occasionally, however, he gives five or ten drops of laudanum; or, when the spasms are violent, forty, but never more. He allows no kind of warm drinks. Blisters and mustard poultices, or leeches may be applied to prevent congestion of the brain.

At the beginning of the Epidemic he lost one patient out of six—towards the close, one out of thirty or forty. This difference is common to all the modes of cure which have been employed.

We give this brief abstract of the treatment of the 'physiological school,' without having room for comments, and disposed to let every reader estimate it for himself. D.

